# Supplementary figures and images for: Anti-Inflammatory Activity of No-Ozone Cold Plasma in Porphyromonas gingivalis Lipopolysaccharide-Induced Periodontitis Rats
Source: Int J Mol Sci. 2024 Jun 3;25(11):6161. doi: 10.3390/ijms25116161 (PMC11172587; doi:10.3390/ijms25116161)

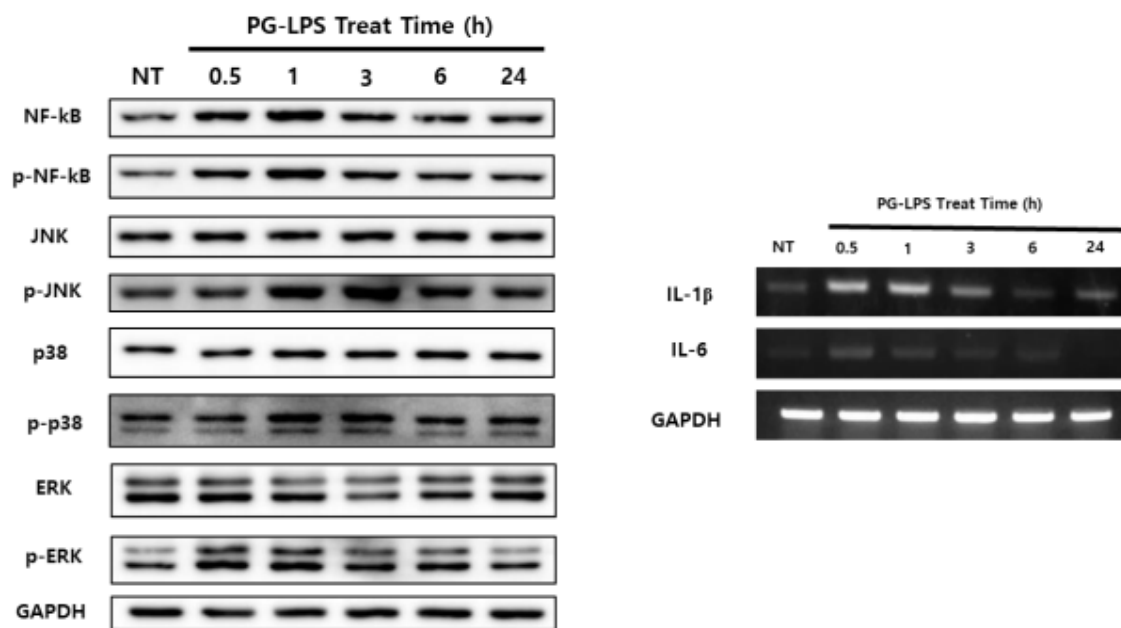

Figure S1. PG-LPS time-dependent.

Supplement: Supplementary file 1 [file ijms-25-06161-s001.zip › ijms-3028007-supplementary.pdf]
